# Comparative Analysis of Human Gut Microbiota by Barcoded Pyrosequencing

**DOI:** 10.1371/journal.pone.0002836

**Published:** 2008-07-30

**Authors:** Anders F. Andersson, Mathilda Lindberg, Hedvig Jakobsson, Fredrik Bäckhed, Pål Nyrén, Lars Engstrand

**Affiliations:** 1 Swedish Institute for Infectious Disease Control, Solna, Sweden; 2 Department of Ecology and Evolution, Limnology, BMC, Uppsala University, Uppsala, Sweden; 3 Department of Microbiology, Cell and Tumor Biology, Karolinska Institutet, Stockholm, Sweden; 4 Department of Molecular and Clinical Medicine, The Sahlgrenska Center for Cardiovascular and Metabolic Research/Wallenberg Laboratory, Göteborg University, Göteborg, Sweden; 5 School of Biotechnology, Albanova, KTH (Royal Institute of Technology), Stockholm, Sweden; Centre for DNA Fingerprinting and Diagnostics, India

## Abstract

Humans host complex microbial communities believed to contribute to health maintenance and, when in imbalance, to the development of diseases. Determining the microbial composition in patients and healthy controls may thus provide novel therapeutic targets. For this purpose, high-throughput, cost-effective methods for microbiota characterization are needed. We have employed 454-pyrosequencing of a hyper-variable region of the 16S rRNA gene in combination with sample-specific barcode sequences which enables parallel in-depth analysis of hundreds of samples with limited sample processing. *In silico* modeling demonstrated that the method correctly describes microbial communities down to phylotypes below the genus level. Here we applied the technique to analyze microbial communities in throat, stomach and fecal samples. Our results demonstrate the applicability of barcoded pyrosequencing as a high-throughput method for comparative microbial ecology.

## Introduction

The human gastrointestinal tract is populated by complex communities of microorganisms, which outnumber the eukaryotic host cells by one order of magnitude [1]. The gut microbiota play important roles in extracting nutrients from the diet [2], [3], regulating host fat storage [4], stimulating intestinal epithelium renewal [5], and directing the maturation of the immune system [6]. Keeping these communities in balance is most likely crucial for health maintenance, and perturbation of microbial composition has been hypothesized to be involved in a range of diseases, within and outside the gut [7], [8]. So far, the most extensive surveys of human microbial ecology have been performed on colonic microbiota (e.g. [9], [10], [11]), whereas less has been reported from upper gastro-intestinal tract habitats (e.g. oral cavity [12], esophagus [13] and stomach mucosa [14]). Although polymerase chain reaction (PCR) amplification, cloning, and sequencing of the 16S ribosomal RNA gene content of microbial samples has revolutionized the characterization of microbial communities [15], [16], this method is expensive and time consuming. Studies have thus been constrained to either include few samples or only describe the dominant members of the communities. Recently developed methods based on microarray technology [17], [18] hold promise for large-scale studies, but they do not capture novel sequences.

In parallel with other groups [19], [20] we have developed a method based on 454-pyrosequencing [21] for monitoring of microbial communities. A highly variable region of the 16S rRNA gene is amplified using primers that target adjacent conserved regions, followed by direct sequencing of individual PCR products. Here we demonstrate the power of this method by exploring the diversity within human gut ecosystems, from throat to colon. We show that the method produces taxonomic classifications of high fidelity when relevant reference 16S rRNA sequences are available. The results confirm previous cloning-based investigations of the gastro intestinal tract and provide novel insights into the throat microbiota.

## Results

### Barcoded 16S pyrosequencing

**Figure 1:**
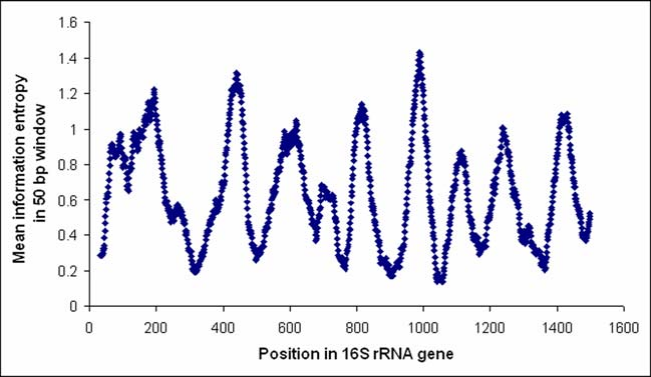
Variability within the 16S rRNA gene. From pre-aligned sequenced >1200 bp downloaded from RDP, the variability, measured as Shannon information entropy, was calculated at each sequence position, using only positions without a gap in *E. coli.* The graph shows the Shannon entropy (y-axis) averaged over 50 bp windows, centered at each position in the gene (x-axis). Shannon entropy at position *x* was calculated as –Σ *p*(*x_i_*) log*_2_ p*(*x_i_*), where *p*(*x_i_*) denotes the frequency of nucleotide *i*. The filled arrows indicate positions of the PCR primers, the dashed arrow the direction of sequencing.

In our setup, a ∼280 nt region of the 16S rRNA gene (*Escherichia coli* position 781 to 1,060) is amplified by PCR. This region, which includes variable region V6, was selected since it displays high variability (Fig. 1) and is surrounded by conserved regions [22], [23]. In order to function well in samples with low bacterial/host cell ratios, primers were selected not to match the human genome, and tested not to render PCR amplification with human DNA as template (data not shown). We included a sample-specific four-nucleotide barcode sequence on one of the primers to allow multiple samples to be analyzed in parallel on a single 454 picotiter plate [18]. Each pyrosequencing read is BLAST [24] searched against a reference database comprising >90,000 near full-length 16S rRNA gene sequences from the Ribosomal Database Project (RDP) [25]. The best matching near full-length sequence that fulfills certain criteria on similarity (Materials and Methods) is selected to represent the pyrosequencing read, and, consequently, the read inherits the taxonomic classification (down to genus level) of the reference sequence.

### *In silico* evaluation

**Figure 2:**
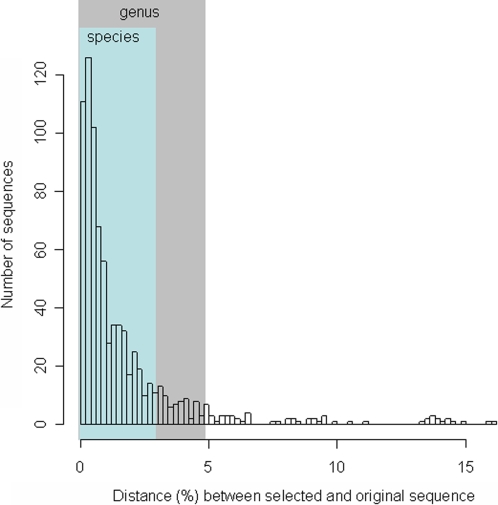
Taxonomic classification accuracy. Distribution of sequence distances (measured over the whole sequence lengths) between original sequence and the selected reference sequence, when 59 bp corresponding to minimal pyrosequencing reads were extracted from 1000 randomly selected RDP sequences and assigned to reference RDP sequences according to the procedure described in the Materials and Methods section (in this case the 1000 sequences had first been removed from the BLAST database).

To evaluate the precision of the method we performed *in silico* modeling using pre-existing near full-length (>1200 bp) sequences of the RDP database. We selected 1000 sequences at random that matched our reverse primer and extracted subsequences downstream the primer corresponding to minimal pyrosequencing reads (59 bp; Materials and Methods). These artificial reads were BLAST searched against the RDP database, from which the corresponding sequences first had been removed. Eighty-one percent of the artificial pyrosequencing reads had approved matches to database sequences. Among these, the reference and original sequence differed on average by 1.7% over the full length of the sequences, and 85% of the pairs displayed <3% difference at the nucleotide level (Fig. 2), a limit typically used to assign bacteria to the same species [26]. Moreover, for 94% of the pairs where query and selected hit were classified down to genus level in RDP, both sequences were classified as the same genus.

### Addressing the effect of sequencing errors on taxonomic classifications

454-pyrosequencing has been reported to have a relatively high homopolymer insertion/deletion error rate [21] which could potentially disturb the taxonomic classifications. To address this issue, we identified all sequences from our pyrosequencing run that could be converted into other sequences in the run by deleting one nucleotide anywhere within the sequences (deleted sequences that were sub-sequences of the original were not considered, i.e. deletions within homopolymers in the beginning or end of sequences). 4,460 unique pairs of sequences related in this way were found. The average ratio between the total number of reads for the more frequent and the less frequent sequence was 201∶1, compared with 16∶1 for 4,460 randomly selected pairs, indicating that the less frequent sequence in many such pairs resulted from sequencing errors (the correct sequence is likely to be much more abundant than the artifact). However, in 92.2% of the pairs both were classified as the same RDP sequence and among the pairs where both RDP representatives were classified down to genus level, 99.5% belonged to the same genus (compared with 1.7% and 5.8% for the random pairs). Thus, although insertion/deletion errors seem to occur to some extent, the application here is robust: an insertion/deletion error rate of 2% of reads [21] would affect the classification of 0.2% of the total number of reads.

### Overview of human gut microbial communities

Here we have applied the method to analyze the microbial ecology of throat, stomach and fecal samples; we analyzed both throat and fecal samples from 6 subjects, and obtained stomach samples from a further 6 subjects (3 negative and 3 positive for *H. pylori* according to culturing). In total, 61,768 reads were captured from the 18 samples. After filtering out reads that contained incorrect primer sequences or were shorter than 80 nucleotides (to leave a minimum of 59 nucleotides downstream of the primer for taxonomic classification), 56,382 reads, with a mean length of 73 nucleotides downstream the primer, remained. An RDP reference sequence could be assigned to 49,514 (88%) of these reads, generating 2,751±1,348 (s.d.) annotated reads per sample. The entire dataset was represented by 911 RDP sequences, which were further clustered into 609 phylotypes with maximum within-cluster dissimilarity of 3% [26].

**Figure 3:**
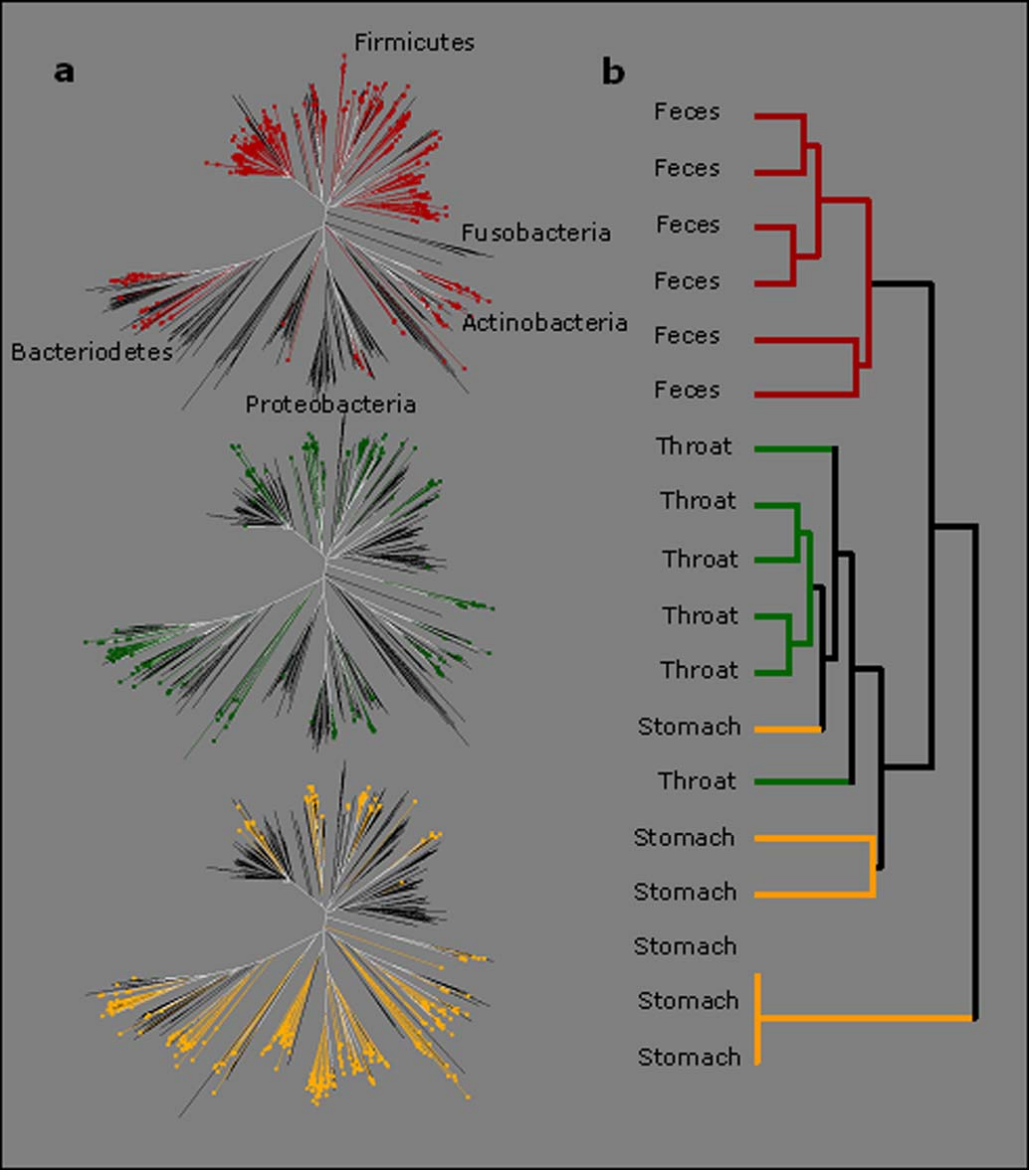
Comparison of the throat, stomach and fecal microbiotas. a, A neighbor joining phylogenetic tree of the RDP sequences representing the 454 reads from six samples of throat, stomach, and feces, respectively, was constructed. Branches in the tree represented in throat, stomach, and feces are labeled with green, yellow, and red, respectively. b, Hierchical clustering of the 18 samples based on how their reads were distributed within the tree using the weighted UniFrac metric [27] for pair wise comparisons of the samples. The lower three samples are *H. pylori* positive stomachs.

To investigate whether we could identify similarities between the microbial populations in the throat, stomach and fecal samples, we constructed a phylogenetic tree based on the RDP sequences representing the pyrosequencing reads (Fig. 3a). The samples were then clustered based on how their reads were distributed within the tree using the UniFrac method [27] (Fig. 3b). We found that the fecal samples formed a distinct cluster while the throat and stomach samples grouped more closely. The three stomach samples that were positive for *H. pylori* by culturing branched separately. The vast majority (>99%) of the annotated reads belonged to five bacterial phyla: Firmicutes, Actinobacteria, Bacteroidetes, Proteobacteria and Fusobacteria (Table 1). Remaining annotated reads belonged to the Spirochaetes, Cyanobacteria, Acidobacteria, Chlamydiae, Gemmatimonadetes, Planctomycetes, Verrucomicrobia, and the uncultivated phyla TM7 and OP10.

**Table 1:** Representation of bacterial phyla within different sample groups.

Percentage of reads (±SD)						
	Firmicutes	Actinobacteria	Bacteroidetes	Proteobacteria	Fusobacteria	Others
Throat (n = 6)	55.6±13.6	14.5±3.9	20.0±8.6	4.7±3.4	5.1±3.7	<1
*H. pylori* negative stomach (n = 3)	29.6±15.9	46.8±18.9	11.1±8.7	10.8±3.2	1.1±1.1	<1
*H. pylori* positive stomach (n = 3)	1.8±0.6	1.1±0.7	0.8±0.6	96.2±1.8	0.1±0.01	<0.1
Feces (n = 6)	81.2±11.2	14.6±9.8	2.5±2.6	1.7±1.5	0	<0.1

The majority of reads that could not be annotated accurately had closest matches to the phyla mentioned above. However, for 47 reads (37 unique sequences of which 29 were found in stomach), the closest matches were from uncultured organisms that had not been placed into recognized phyla in RDP (Table S2), and might thus represent bacterial divisions not yet described. Only 107 reads (0.2%) had best BLAST hits of <90% identity to any RDP sequence (Table S2).

To get an estimate of how quantitative the method is, an artificial sample was analyzed consisting of a mixture of three bacterial strains, two Gram-negative and one Gram-positive. Similar amounts of cells, as measured by viable counts, of *H. pylori*, *E. coli* and *Streptococcus pyogenes* were added before DNA extraction. The number of reads correlated approximately with the number of encoded 16S rRNA genes; 306 reads and 2 operons in *H. pylori*, 478 reads and 6 operons in *S. pyogenes,* and 828 reads and 7 operons in *E. coli*.

### A well defined throat community

**Figure 4:**
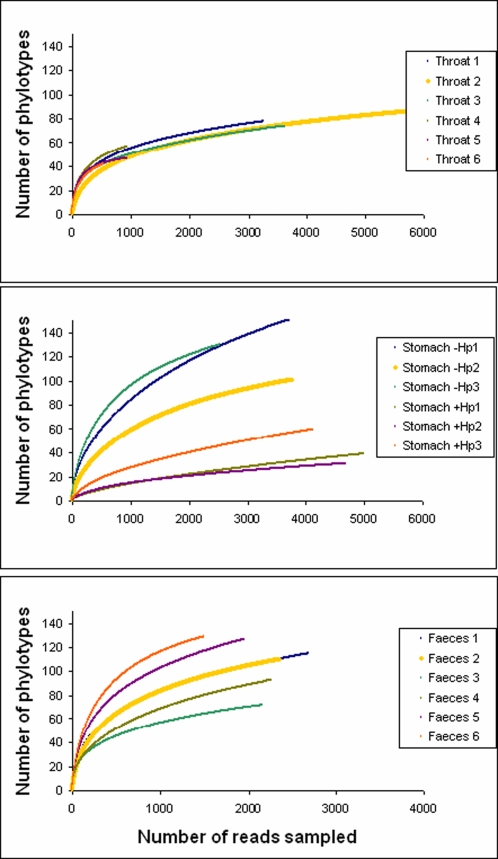
Rarefaction analysis of the different gut ecosystems. Number of phylotypes sampled as a function of number of reads. The data points represent averages of 1000 randomized samplings without replacements.

The throat microbiota displayed the lowest phylotype richness of the three ecosystems (Fig. 4, for diversity estimates see Table 2), with 152 phylotypes of which 20 represented 90% of the reads. It also showed the highest similarity between individuals (Fig. 4, for pairwise sample comparisons see Fig. S1), indicating a highly stable microbial community. The microbiota was similar to that of the distal esophagus reported earlier [13]. Eight genera (*Streptococcus, Prevotella, Actinomyces, Gemella, Rothia, Granulicatella, Haemophilus* and *Veillonella*) were present in all of our throat samples and in the previously reported esophagus samples, and constituted >75% of the total sequences in both communities. At both sites, *Streptococcus* was the dominant genus followed by *Prevotella*. A differentiating genus was *Veillonella*, representing 14% of the esophagus sequences but only 0.4% of the throat reads.

**Table 2:** Estimations of diversity within different sample groups.

	Number of reads	Number of OTUs	Chao1 estimated richness	Shannon diversity index	Rao diversity coefficient	Good's estimated coverage)
Throat (n = 6)	13035	152	204	2.64	0.199	99.7%
*H. pylori* negative stomach (n = 3)	9958	262	375	3.01	0.222	99.1%
*H. pylori* positive stomach (n = 3)	13755	85	128	0.305	0.024	99.7%
Feces (n = 6)	12766	301	385	4.03	0.19	99.4%

Feces displayed the highest diversity as measured by the Shannon index, which only considers relative phylotype abundances. According to the Rao coefficient, which also takes phylotype dissimilarities into account, the uninfected stomach harboured highest diversity. Good's estimated coverage shows that throat samples and *H. pylori* infected stomachs are most completely sampled, where one new phylotype would be expected per 341 additional reads.

### A diverse stomach microbiota

Our analysis revealed diverse microbial communities in the three *H. pylori*-negative stomachs. These harbored 262 phylotypes representing 13 phyla, including reads from phyla not detected in the stomach previously, e.g. Chlamydia (10 reads) and Cyanobacteria (6 reads). Our results corroborate the finding that the stomach displays a diverse microbiota when *H. pylori* is absent or low in abundance [14]. To what extent this represents resident or transient populations of ingested microbes is unclear. However, only 33 phylotypes were found in all three *H. pylori-*negative samples and most of the prominent phylotypes (e.g. *Streptococcus, Actinomyces, Prevotella* and *Gemella*) were also abundant in the throat, suggesting that they may represent swallowed microorganisms from upstream microbiota. High inter-subject variability was observed even for abundant taxa: the genus *Rothia* dominated one of the samples (60% of reads) but constituted only 3.6% of another sample; this second sample was dominated (24% of reads) by *Bifidobacterium*. The majority of the 177 phylotypes found in stomach but not in throat belonged to the Proteobacteria.

Strikingly, the three samples that were positive for *H. pylori* by culturing were totally dominated by this bacterium, comprising 93–97% of the reads, thus dramatically reducing the diversity (Fig. 4). These findings indicate how well this bacterium is adapted to the stomach habitat. The pyrosequencing analysis revealed that different *H. pylori* strains dominated the three samples; the dominant sequence of one of the samples had a single bp substitution relative to the others'. The dominance of *H. pylori* was more pronounced than in a recent study [14], where 72% of the sequences in the *H. pylori* positive samples were derived from this species. The difference may potentially reflect inter-subject variability, or differences in sampling procedures.

### Abundant Actinobacteria in the lower intestine

The human lower intestine is the most densely populated microbial ecosystem known, with approximately 10^12^ microorganisms/ml [1], and is considered to be dominated by the phyla Firmicutes and Bacteriodetes [9], [10], [11]. In our pyrosequencing analysis, Firmicutes dominated the six fecal samples with 235 phylotypes and >80% of the reads (Table 1). The majority of the Firmicutes (92±6%) belonged to the class Clostridia with frequent representation of the genera *Ruminococcus*, *Clostridium* and *Eubacterium.* Surprisingly, Actinobacteria was the second most abundant phylum in all samples (Table 1), significantly outnumbering the Bacteroidetes (*t* test *P* = 0.025). The Actinobacteria were dominated by a few phylotypes belonging to the genus *Bifidobacterium* (8±7% (s.d.) of reads) and to the family Coriobacteriaceae (6±3% (s.d.)) while the Bacteroidetes were dominated by various *Bacteroides* phylotypes.

## Discussion

In our approach we match the pyrosequencing reads to full-length, taxonomically classified, reference 16S rRNA sequences, based on sequence similarities deduced by BLAST. This works well when highly similar sequences are present in the database (identical or differing by a few bases). When analyzing less well characterized communities, many reads will lack close matches. For these, taxonomic classification will be restricted to higher phylogenetic levels, which may be more accurately done using other methods [28].

Among the sequence reads obtained here only 0.2% had best BLAST hits of <90% identity to any RDP sequence. This contrasts sharply with a recent survey of the deep sea microbiota [19] using 454-pyrosequencing, where >25% of the reads displayed >10% divergence from existing sequences. The discrepancy likely reflects the richer representation of gut sequences within current 16S databases, and also the much higher diversity of the deep sea microbiota, which has evolved and diversified in a habitat that has persisted over billions of years [19].

Interestingly, Actinobacteria were more abundant than Bacteriodetes in all six fecal samples analyzed, contrasting with prior studies. It is possible that Bacteroidetes are under-represented in our six fecal samples, because this phylum is known to show inter-subject variability [9], to vary in response to adiposity [10] and to sometimes be suppressed in inflammatory bowel disease [11]. Notably, the samples analyzed here derive from subjects older than those of previous extensive 16S surveys [9], [10], and culture-based studies have shown a decline of Bacteriodetes with increasing age [29]. However, discrepancies may partly be explained by PCR biases; a comparison with the RDP database shows that the primers used here are significantly more sensitive for Actinobacteria than commonly used primers (Table S1).

Even though the primers used here have improved range compared to frequently used 16S primers (Table S1), they are not universal for the domain bacteria, and hence sequences will remain undetected. Primer sequences can likely be further improved; a complicating factor is however the risk of amplifying human DNA, which considerably restricts the choice of primers. Other potential sources of errors in the methodology are sequence-specific PCR amplification differences and biases introduced by DNA extraction. As indicated by our results, rRNA operon copy variation should also be taken into account when estimating bacterial abundances. However, well designed studies with cases and controls should reveal imbalances among microbial taxa, even though absolute abundances remain unknown.

The recent demonstration that obesity is reflected in the intestinal microbial composition in both mice [30] and humans [10], and that the obesity trait is transmissible through transplantation of the microbiota [3] clearly illustrates how the microbial community can effect host physiology. To investigate whether other diseases are associated with, or caused by, changes in the microbial gut ecology, large-scale, well-designed epidemiological studies are needed. The high-throughput methodology demonstrated here provides a means for such studies.

## Materials and Methods

### Samples

Stomach biopsies were obtained by upper endoscopy of gastric corpus from six healthy individuals (aged 61–76 years) who were part of a randomized population-based study on peptic ulcer disease [31]. Of the six biopsies three were *H. pylori* positive by culture. The biopsies were placed in freezing medium with 10% glycerol and frozen immediately at −20°C after the endoscopy, and moved to −70°C within 2 weeks. The study was approved by the ethics committee of Umeå University, Sweden, May 29, 1998. Fecal samples and throat swabs were collected from three patients (aged 42–73 years) with duodenal ulcer and three dyspeptic controls (aged 70–75 years) who were part of a longitudinal cohort study [32]. All samples arrived at the laboratory within 24 h and were stored at −70°C. The study was approved by the ethics committee at Uppsala University, Sweden, June 10, 1997.

### DNA extraction

For total genomic DNA extraction of stomach biopsies, samples were homogenised by a pestle (2×10 s) in 1.5 ml tubes with 500 µl freezing medium. The homogenate (100 µl) was lysed in 180 µl lysozyme buffer (20 mM Tris-HCI, pH 8.0, 2 mM sodium EDTA, 1.2% Triton X-100, and 20 mg/ml lysozyme (Sigma-Aldrich, Schnelldorf, Germany)) and incubated at 37°C for 1 h. Proteinase K and 200 µl Buffer AL were added and the mixture was incubated for another 16 h at 56°C followed by Qiageńs DNeasy Tissue Kit (Qiagen, Hilden, Germany). Samples were eluted in 100 µl Buffer AE. A negative extraction control without sample was also included. To extract DNA from throat swabs, 250–500 µl samples were diluted (1∶1) in a dilution buffer (20 mM Tris-HCI and 2 mM EDTA (pH 8.0)) and centrifuged for 10 min at 5000×g. The procedure was then as described for the stomach biopsies. DNA was extracted from 100 mg feces using a FastDNA SPIN Kit for Soil (BIO 101, Carlsbad, CA) according to the manufacturer's instructions. The bead-beating step was performed in a FastPrep Instrument for 2×20 s at speed 5.5.

For the artificial sample *H. pylori* CCUG 47164, *E. coli* ATCC 25922 and *S. pyogenes* ATCC 12344 were individually grown to OD_600_ = 0.3. 5×10^4^. Similar amounts of cells, according to viable counts, of each strain were pooled and DNA was extracted as described above for stomach biopsies and throat.

### Primer design

To function as broadly as possible for characterizing human-associated microbiotas, a primer pair was designed based on the following criteria: 1) the amplified region should be highly variable enabling discrimination between closely related taxa; 2) the primers should be present in a large proportion of known 16S rRNA sequences (see Table S1 for data on primer coverage); and 3) the primers should not yield substantial PCR product using human genomic DNA as template. Based on these criteria, a primer pair was designed that amplifies *E. coli* position 981 to 1,060 of the 16S rRNA gene, which includes the highly variable region V6. The forward primer (784F) carried the 454-adaptor sequence-B in the 5′ end, and the reverse primer (1061R) 454-adaptor sequence-A in the 5′ end, followed by a sample specific barcode sequences (Table 3).

**Table 3:** Primer, adaptor and sample-specific barcode sequences.

Primer	Adaptor sequence	Barcode sequence	Primer sequence
784F	GCCTTGCCAGCCCGCTCAG		AGGATTAGATACCCTGGTA
1061R_1	GCCTCCCTCGCGCCATCAG	CGAT	CRRCACGAGCTGACGAC
1061R_2	GCCTCCCTCGCGCCATCAG	CATG	CRRCACGAGCTGACGAC
1061R_3	GCCTCCCTCGCGCCATCAG	CTGA	CRRCACGAGCTGACGAC

The reverse primers have two degenerate nucleotide positions where R denominates A/G.

The sequencing reaction is primed by an oligonucleotide complementary to the adaptor sequence of the reverse primer, such that the barcode sequence will be read first, followed by the primer sequence, followed by the variable 16S rDNA sequence.

### PCR, template preparation and sequencing

For each sample, a 50 µl PCR mix was prepared containing 1×PCR buffer, 200 µM dNTP PurePeak DNA polymerase Mix (Pierce Nucleic Acid Technologies, Milwaukee, WI,), 0.5 µM of each primer (SGS, Köping, Sweden) and 2.5 U PfuUltra High-Fidelity DNA polymerase (Stratagene La Jolla, CA). To each reaction 1–10 µl of the extracted template-DNA was added. The PCR conditions used were 95°C for 5 min, 30 cycles of 95°C for 40 s, 55°C for 40 s and 72°C for 1 min, followed by 72°C for 7 min. The negative extraction control was amplified with 35 cycles in the PCR.

The PCR products, with approximate length of 270 nt, were excised from the agarose gel (1% in TBE buffer) containing ethidiumbromide, and purified with QIAquick gel extraction kit (Qiagen, Hilden, Germany). The DNA concentration and quality were assessed on a Bioanalyzer 2100 (Agilent, Palo Alto, CA) using a DNA1000 lab chip (Agilent, Palo Alto, CA). Equal amounts of three samples with different sample-specific barcode sequences were pooled to a total amount of 100 ng. The pooled DNA were subsequently amplified in PCR-mixture-in-oil emulsions and sequenced on different lanes of a 16-lane PicoTiterPlate on a Genome Sequencer 20 system [21] (Roche, Basel, Switzerland) at 454 Life Sciences (Branford CT) in June 2006. The negative control was sequenced on an individual lane. Reads in the samples also present in the negative control were excluded from further analysis.

### Taxonomic classification of sequence reads

90,211 16S rDNA sequences longer than 1,200 bp were downloaded from RDP v. 9.39 and formatted into a local BLAST database. Since 59 bp was sufficient for classification, and since the number of reads sharply dropped for reads shorter than 80 bp, all pyrosequencing reads of length ≥80 bp containing a correct primer sequence, and without ambiguous bases, were extracted and cured from primer/barcode sequence (leaving a minimum of 59 bp for taxonomic classification). Each resulting unique sequence (one per group of identical sequences) was BLASTN-searched against the RDP database with default parameters. The best scoring hit was selected to represent the pyrosequencing sequence if it displayed ≥95% identity (mean = 0.996 for approved reads) over an alignment of length ≥[query length–5 bp].

If multiple best scoring hits fulfilled these criteria, the most representative sequence was selected by the following procedure: The average sequence distance (over the length of the whole sequences) between each hit and the other best scoring hits was calculated based on a distance matrix generated in ARB [33]. The sequence with lowest average distance to the other hits was selected if its average distance was below 0.04; otherwise the pyrosequencing sequence was excluded from further analysis. *In silico* evaluations suggested this selection procedure to be effective, in part because it reduced the risk of selecting chimeric sequences as references (data not shown).

### Calculating sequence distances and grouping into phylotypes

RDP sequences rendering best scoring BLAST hits to the pyrosequencing reads, as well as *E. coli* sequence S000380829, were downloaded in pre-aligned format from RDP and imported into ARB[33]. A pair-wise distance matrix was generated employing Olsen correction and masking nucleotides not present in the *E. coli* sequence (since the RDP alignment was based on an *E. coli* sequence). The distance matrix was imported into DOTUR [26] to cluster the RDP sequences into phylotypes (OTUs) of maximum within-cluster dissimilarity (furthest neighbor) of 3%. The RDP sequence with the highest number of corresponding pyrosequencing reads, in the entire dataset, was selected to represent each phylotype.

### Phylogenetic tree construction and sample clustering

A neighbor-joining phylogenetic tree of the selected RDP representative sequences was constructed in ARB, employing Olsen correction. The online version of UniFrac (http://bmf.colorado.edu/unifrac) was used to calculate weighted (incorporating abundance data) UniFrac distances between the samples. Samples were clustered (unweighted pair-group average method) using the *R* software (http://www.r-project.org/).

### Diversity estimations

A Perl script was written for rarefaction analysis (random sampling without replacement, average of 1000 iterations), sampling coverage and diversity estimations. Good's coverage estimation was calculated as [1–(n/N)]×100, where n is the number of singleton phylotypes and N is the number of sequences [34]; Shannon diversity index as –Σ log(p*_i_*)p*_i_*, were p*_i_* denotes the frequency of phylotype *I* [35]; Rao diversity coefficient as ΣΣ p*_i_* p*_j_* d*_ij_*, where d*_ij_* is the dissimilarity between sequence *i* and *j* [36]; Bias–corrected Chao1 estimation of species richness as S_obs_+f_1_(f_1_–1)/f_2_(f_2_–1), where S_obs_ is the number of observed phylotypes and f_1_ and f_2_ the frequencies of singleton and doubleton phylotypes, respectively [37].

## Supporting Information

Figure S1 (0.12 MB DOC)

Table S1 (0.06 MB DOC)

Table S2 (0.04 MB XLS)
