# Supplementary material for: Comparative Analysis of Human Gut Microbiota by Barcoded Pyrosequencing
Source: PLoS One. 2008 Jul 30;3(7):e2836. doi: 10.1371/journal.pone.0002836 (PMC2475661; doi:10.1371/journal.pone.0002836)
Supplement: Figure S1 — (0.12 MB DOC) [file pone.0002836.s001.doc]

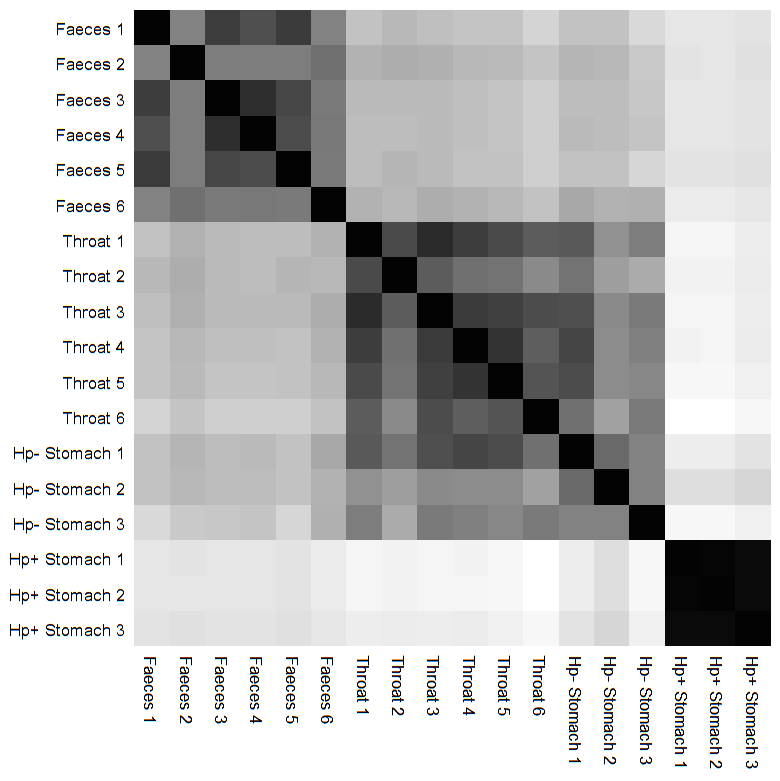

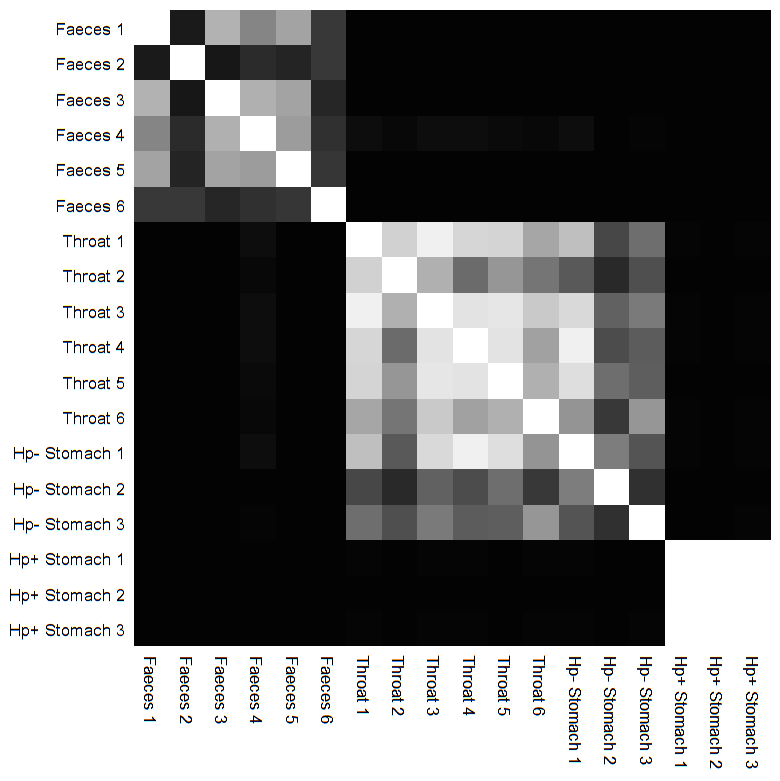


a

b


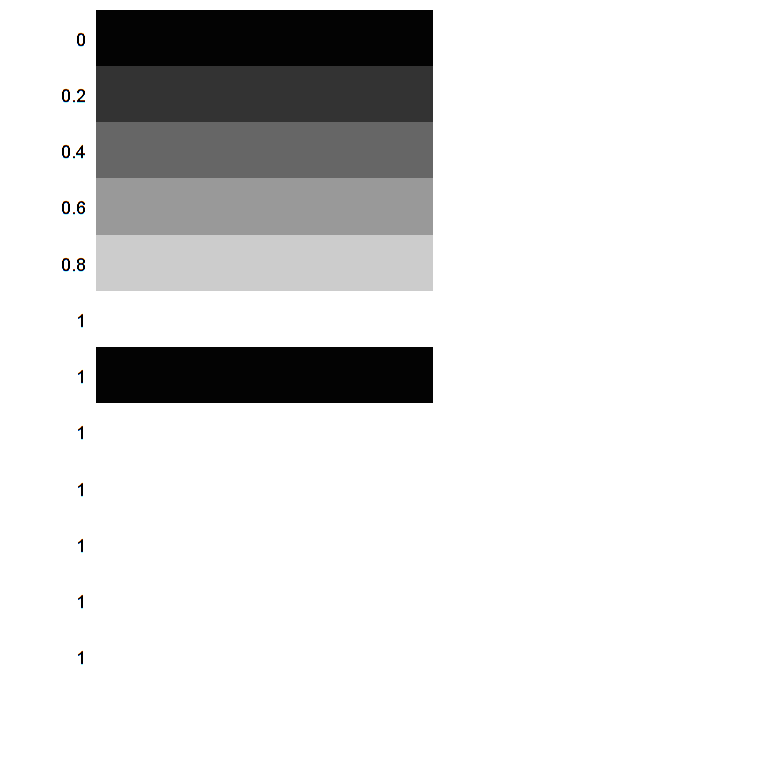


Fig. S1: Pairwise sample comparisons. Each sample was compared to one another by using a) weighted UniFrac metric (UF; between 0 for communities sharing all, and 1 for communities not sharing any branches of a combined phylogenetic tree), and b) Pearson correlation between phylotype counts (*r^2^*; between 0 for no, and 1 for perfect correlation). The within sample-type averages were: Throat: UF = 0.096 and *r^2^* = 0.56; *H. pylori* negative stomach: UF = 0.14 and *r^2^* = 0.13; *H. pylori* infected stomach: UF = 0.0090 and *r^2^* = 1.00; Faeces: UF = 0.12 and *r^2^* = 0.18.
