# Supplementary material for: Comparative Analysis of Human Gut Microbiota by Barcoded Pyrosequencing
Source: PLoS One. 2008 Jul 30;3(7):e2836. doi: 10.1371/journal.pone.0002836 (PMC2475661; doi:10.1371/journal.pone.0002836)
Supplement: Table S1 — (0.06 MB DOC) [file pone.0002836.s002.doc]

| **Phylum** | **Total** | **784F** | **1061R** | **8F** | **8Fmod** | **1392R** | **1510R** |
| --- | --- | --- | --- | --- | --- | --- | --- |
| Acidobacteria | 3 | 3 | 3 | 1 | 1 | 3 | 0 |
| Actinobacteria | 83 | 82 | 83 | 42 | 52 | 83 | 0 |
| Aquificae | 2 | 0 | 2 | 2 | 2 | 2 | 0 |
| Bacteroidetes | 27 | 27 | 27 | 22 | 22 | 27 | 0 |
| Chlamydiae | 13 | 13 | 13 | 0 | 0 | 13 | 0 |
| Chlorobi | 5 | 5 | 5 | 3 | 3 | 5 | 0 |
| Cyanobacteria | 36 | 0 | 36 | 21 | 21 | 32 | 0 |
| Deferribacteres | 0 | 0 | 0 | 0 | 0 | 0 | 0 |
| Deinococcus-Thermus | 11 | 0 | 11 | 7 | 7 | 11 | 0 |
| Firmicutes | 505 | 489 | 501 | 347 | 347 | 504 | 0 |
| Fusobacteria | 5 | 5 | 5 | 5 | 5 | 5 | 0 |
| Genera_incertae_sedis_Dehalococcoides | 2 | 0 | 2 | 0 | 0 | 2 | 0 |
| Proteobacteria | 847 | 824 | 847 | 210 | 584 | 809 | 218 |
| Spirochaetes | 15 | 7 | 15 | 8 | 8 | 15 | 0 |
| Thermotogae | 1 | 0 | 1 | 0 | 0 | 1 | 0 |
|  |  |  |  |  |  |  |  |
| Total | 1555 | 1455 | 1551 | 668 | 1052 | 1512 | 218 |

**Table S1: Primer coverage.** Primer sequences were compared with RDP 16S rRNA gene sequences encoded on sequenced bacterial genomes and exact matches were counted. Primer 784F (used in this study) has the sequence AGGATTAGATACCCTGGTA;
1061R (used in this study), CRRCACGAGCTGACGAC;

8F, AGAGTTTGATCCTGGCTCAG;

8Fmod, AGAGTTTGATYMTGGCTCAG;

1392R, GACGGGCGGTGTGTRCA and

1510R, CGGTTACCTTGTTACGACTT,

where R = A/G, Y = C/T and M = A/C.
